# Intraoperative B-Mode Ultrasound Guided Surgery and the Extent of Glioblastoma Resection: A Randomized Controlled Trial

**DOI:** 10.3389/fonc.2021.649797

**Published:** 2021-05-19

**Authors:** Fatih Incekara, Marion Smits, Linda Dirven, Eelke M. Bos, Rutger K. Balvers, Iain K. Haitsma, Joost W. Schouten, Arnaud J. P. E. Vincent

**Affiliations:** ^1^ Department of Neurosurgery, Erasmus MC - University Medical Center Rotterdam, Rotterdam, Netherlands; ^2^ Department of Radiology and Nuclear Medicine, Erasmus MC - University Medical Center Rotterdam, Rotterdam, Netherlands; ^3^ Department of Neurology, Leiden University Medical Center, Leiden, Netherlands; ^4^ Department of Neurology, Haaglanden Medical Center, The Hague, Netherlands

**Keywords:** glioblastoma, extent of resection, intraoperative ultrasound, randomized controlled trial, image guided neurosurgery

## Abstract

**Background:**

Intraoperative MRI and 5-aminolaevulinic acid guided surgery are useful to maximize the extent of glioblastoma resection. Intraoperative ultrasound is used as a time-and cost-effective alternative, but its value has never been assessed in a trial. The goal of this randomized controlled trial was to assess the value of intraoperative B-mode ultrasound guided surgery on the extent of glioblastoma resection.

**Materials and Methods:**

In this randomized controlled trial, patients of 18 years or older with a newly diagnosed presumed glioblastoma, deemed totally resectable, presenting at the Erasmus MC (Rotterdam, The Netherlands) were enrolled and randomized (1:1) into intraoperative B-mode ultrasound guided surgery or resection under standard neuronavigation. The primary outcome of this study was complete contrast-enhancing tumor resection, assessed quantitatively by a blinded neuroradiologist on pre- and post-operative MRI scans. This trial was registered with ClinicalTrials.gov (NCT03531333).

**Results:**

We enrolled 50 patients between November 1, 2016 and October 30, 2019. Analysis was done in 23 of 25 (92%) patients in the intraoperative B-mode ultrasound group and 24 of 25 (96%) patients in the standard surgery group. Eight (35%) of 23 patients in the intraoperative B-mode ultrasound group and two (8%) of 24 patients in the standard surgery group underwent complete resection (p=0.036). Baseline characteristics, neurological outcome, functional performance, quality of life, complication rates, overall survival and progression-free survival did not differ between treatment groups (p>0.05).

**Conclusions:**

Intraoperative B-mode ultrasound enables complete resection more often than standard surgery without harming patients and can be considered to maximize the extent of glioblastoma resection during surgery.

## Introduction

Patients with glioblastoma have a poor prognosis with a median overall survival of 15 months, despite surgical resection with concomitant and adjuvant chemoradiotherapy (1). Complete resection of contrast-enhancing tumor on T1-weighted post-contrast MRI has consistently been associated with longer overall survival (2, 3). It is shown that intraoperative technologies, specifically 5-aminolevulinic acid or intraoperative MRI guided surgery, are useful to maximize tumor resection during glioblastoma surgery (4–6). Although intraoperative MRI has been associated with higher rates of complete glioblastoma resection, its use is expensive and time-consuming (6).

Intraoperative ultrasound guidance is used during glioblastoma surgery as a time- and cost-effective intraoperative imaging alternative (7). Retrospective studies have shown that intraoperative B-mode ultrasound has the potential to support the surgeon to maximize the extent of glioblastoma resection (8–10). In addition, advanced ultrasound techniques such as contrast enhanced ultrasound, Doppler and elastopgraphy have the potential to better identify residual tumor volumes during glioma surgery (11–14). As Jenkinson et al. showed in a Cochrane review however, the value of intraoperative ultrasound to maximize tumor resection has never been assessed in a randomized controlled trial (4).

We therefore initiated the first randomized controlled trial assessing the value of intraoperative B-mode ultrasound guided surgery on the extent of glioblastoma resection.

## Materials and Methods

In this randomized controlled trial, patients of 18 years or older with a newly diagnosed, contrast-enhancing presumed glioblastoma, deemed totally resectable, presented at the Erasmus MC (Rotterdam, The Netherlands) were enrolled. Exclusion criteria were tumors located in the basal ganglia, cerebellum, brain stem or crossing the midline thereby prohibiting complete resection; multifocal tumors; patients with a Karnofsky performance status < 60 or with pre-existing neurological deficits (e.g. aphasia, hemiparesis). The study was approved by the Medical Ethical Committee of Erasmus MC (MEC-2015-46). All patients gave written informed consent prior to participation. This trial was reported following the CONSORT guidelines and registered with ClinicalTrials.gov (NCT03531333).

### Randomization and Intervention

We randomly assigned patients (1:1) into intraoperative B-mode ultrasound guided surgery (intervention) or resection under standard neuronavigation (control). Randomization was done *via* www.sealedenvelope.com with use of random computer-generated blocks of four by a research assistant who was not otherwise involved with this study. Neurosurgeons and patients were not blinded for treatment allocation. The primary outcome assessor, an independent neuroradiologist, was blinded for treatment group allocation.

Intraoperative ultrasound guidance was performed with the BK Medical Flex Focus 800 ultrasound system alone or integrated with a neuronavigation system (Brainlab, Munich, Germany). The BK Medical craniotomy 8862 transducer was used, which is a convex array transducer with a sector angle of 66° and a contact surface of 29 x 6mm. B-mode, 2-D ultrasound imaging was used without additional usage of advanced ultrasound modalities such as 3-D imaging, contrast-enhanced imaging or elastography. Intraoperative ultrasound was used before opening of the dura to locate the tumor, during tumor resection and to locate any residual tumor in the surgical cavity. Resection was continued until no residual tumor suspected, hyperechoic lesion as seen on ultrasound images was observed in the surgical cavity, or until further resection was deemed unsafe.

Standard surgery was performed with conventional neurosurgical techniques, such as neuronavigation system, cavitational ultrasonic surgical aspiration and surgical microscope. After wound closure, surgeons were asked in both treatment groups to estimate whether complete tumor resection was achieved (yes or no). Surgery time was measured from skin incision to wound closure. Standard adjuvant chemo-and or radiotherapy and clinical follow-up with periodic MRI scans were followed for patients in both groups (15).

### Outcome Measures

The primary outcome of this study was complete resection of contrast-enhancing tumor on early postoperative MRI. All patients underwent 1.5T or 3T MRI scanning with and without gadolinium-based contrast agent one day before surgery and within 48 hours after surgery. One blinded, independent, highly experienced neuroradiologist assessed the tumor localization and extent of tumor resection by volumetrically measuring initial and residual contrast-enhancing tumor volumes. First, pre- and post-operative T1-weighted contrast scans were loaded into Brainlab Elements. Using the SmartBrush tool, semi-automatic tumor assessment of all tumor involved contrast enhancement on preoperative scans and on post-operative scans (excluding small vessels or blood in the surgical cavity) was performed. Tumor localization in terms of eloquence was rated following the Sawaya classification (grade 1: non-eloquent, grade 2: near eloquent, grade 3; eloquent) (16). Complete resection was defined as ≥ 99% resection of contrast-enhancing tumor volume.

Secondary outcomes were: extent of tumor resection (%); neurological status on the National Institutes of Health Stroke Scale (NIHSS) within one week after surgery; functional status on Karnofsky performance scale seven weeks, three months and six months after surgery; change over time in health-related quality of life (EORTC QLQ-C-30 (10) and QLQ-BN20 (17–19) questionnaire) from baseline up to six months after surgery; complication rates; overall survival and progression free survival. EORTC scoring procedures were followed to calculate scale scores (20). Three QLQ-C30 scales (global health, physical functioning, cognitive functioning) and two QLQBN-20 scales (motor dysfunction and communication deficits) were preselected for analysis. A change over time of ≥10 points were classified as clinically meaningful changes (21). Complications were classified according to the US National Cancer Institute common toxicity criteria (CTCAE, version 4.0). Overall survival was defined as time from surgery to death and progression-free survival was defined as time from surgery till clinical or radiological progression following the RANO criteria (22).

### Statistical Analysis

Sample size calculation was performed for the primary outcome based on retrospective studies on intraoperative ultrasound and standard surgery as described in the trial protocol (**Supplemental Material**). Based on complete resection rates of the conventional treatment arm as reported by Stummer et al. we estimated that in the standard surgery group 36% of patients would have complete tumor resection (5). With an estimated effect size of 40% increase of complete resection proportion, power of 80% and significance level at 0.05, we calculated that each group had to include 23 patients. To account for the possibility of drop-out or missing data, we increased the sample size to 25 patients per treatment arm and a total of 50 patients.

Statistical analyses were performed with SPSS 25.0 statistical software (IBM Corp.). Descriptive statistics were tested between treatment arms with the Chi Squared test or Fisher Exact test in case of categorical variables and with the Mann-Whitney U test in case of continuous non-normal distributed data. Survival data were compared between treatment groups with log rank tests and Kaplan Meier estimates and analyzed with multivariable Cox proportional-hazards models. Linear Mixed Models were used to compare health-related quality of life scores over time between treatment arms.

## Results

**Figure 1:**
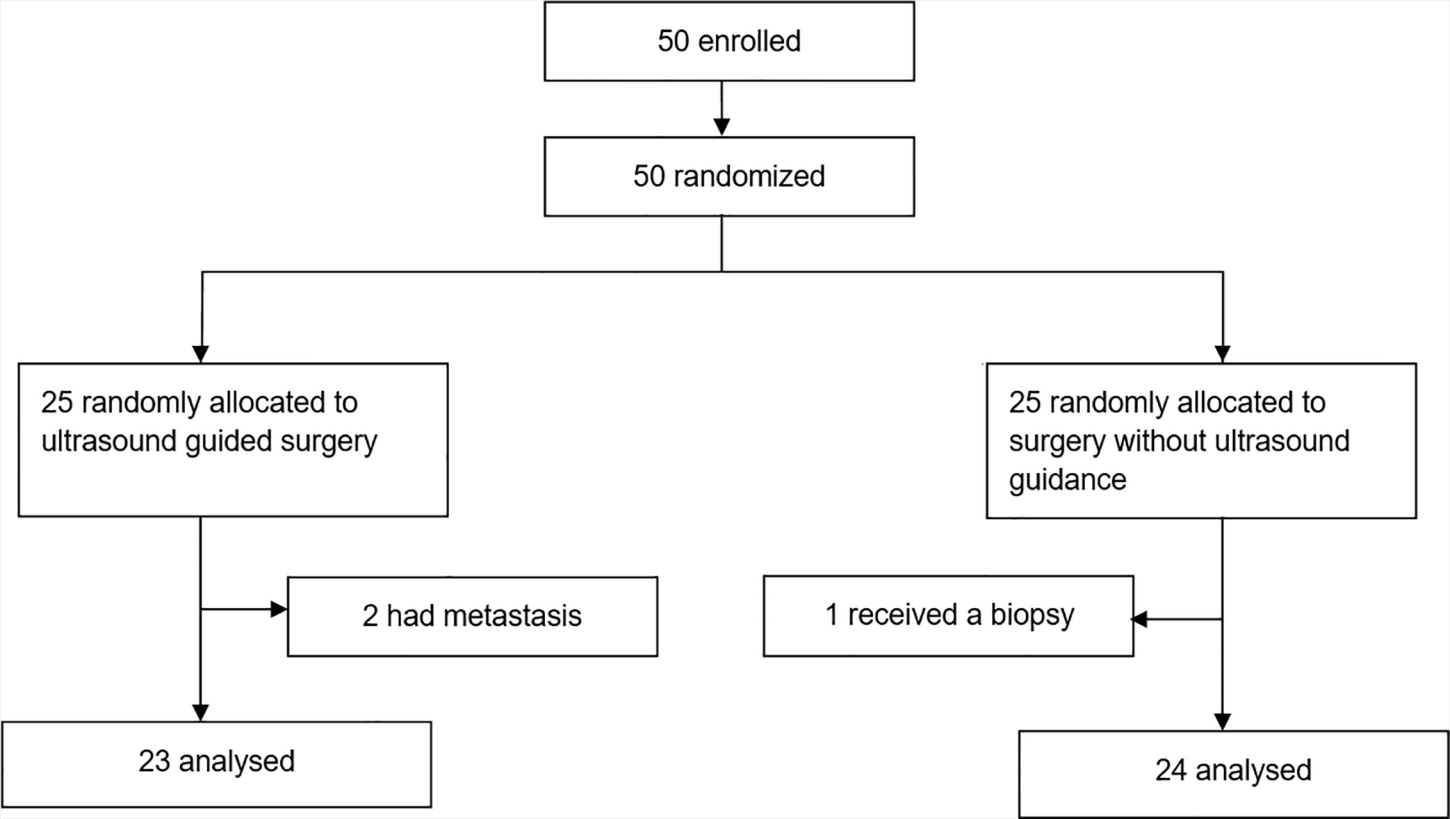
Flowchart.

We enrolled 50 patients between November 1, 2016 and October 30, 2019. Two patients who were diagnosed with metastases after surgery in the ultrasound group and one patient who received a biopsy instead of surgery in the control group were excluded from all further analyses (**Figure 1**). Patient and tumor baseline characteristics did not differ between treatment groups (**Table 1**). Eight (35%) of 23 patients in the intraoperative ultrasound group and two (8%) of 24 patients in the standard surgery group had complete resection of contrast-enhancing tumor (odds ratio 5.9 (95% CI 1.1-31.6), p=0.036; proportion difference 27% (95% CI, 2.8-47.7), p=0.024). Median extent of resection was 97% (IQR 89-100) with intraoperative ultrasound and 95% (IQR 79-98) with standard surgery (p=0.151, **Table 2**). Median residual tumor volume was 0.9 cm^3^ (IQR 0.2-3.4) with intraoperative ultrasound and 1.4 cm^3^ (IQR 0.7-6.4) with standard surgery (p=0.205). Patient outcome of both treatment groups are presented in **Table 3**.

**Table 1:** Baseline characteristics.

	Intraoperative ultrasound (n=23)	Resection under standard neuronavigation (n=24)
Age, median years (IQR)	62 (54-71)	64 (57-70)
Sex		
Male	14 (61%)	14 (58%)
Female	9 (39%)	10 (42%)
KPS, median (IQR)	90 (80-100)	90 (80-100)
Tumor localization*		
Non-eloquent	8 (35%)	8 (33%)
Near eloquent	6 (26%)	6 (25%)
Eloquent	9 (39%)	10 (42%)
Tumor volume, median cm^3^ (IQR)	38.6 (16.9-60.1)	32.3 (17.2-44.6)
NIHSS, median (IQR)	1 (0-1)	0 (0-2)
Quality of life, mean (SD)**		
Global health status	75 (24)	77 (17)
Physical functioning	88 (15)	91 (16)
Cognitive functioning	88 (16)	85 (21)
Motor dysfunction	12 (18)	10 (21)
Communication deficit	17 (24)	9 (14)

Data are No. (%), unless stated otherwise. *Sawaya Grading System **For global health status, physical functioning and cognitive functioning, a higher score represents better functioning. For motor dysfunction and communication deficit, a higher score represents more problems.

KPS, Karnofsky performance status; IDH, isocitrate dehydrogenase; MGMT, methylguanine; DNA, methyltransferase; NIHSS, National Institutes of Health stroke score.

**Table 2:** Surgery outcome.

	Intraoperative ultrasound (n=23)	Resection under standard neuronavigation (n=24)	p value
Resection			0.036*
Complete	8 (35%)	2 (8%)	
Incomplete	15 (65%)	22 (92%)	
Extent of resection, median (IQR), %	97 (89-100)	95 (79-98)	0.151
Residual tumor volume, median (IQR), cm^3^	0.9 (0.2-3.4)	1.4 (0.7-6.4)	0.205
Surgery time, median (IQR), minutes	177 (135-255)	179 (146-227)	0.907
Blood loss, median (IQR), ml	150 (0-400)	125 (58-200)	0.729

Data are n or n (%), unless stated otherwise. *Significant, p value <0.05.

**Table 3:** Patient outcome.

	Intraoperative ultrasound (n=23)	Resection under standard neuronavigation (n=24)	p value
IDH mutation			0.494
Mutated	0 (0%)	0 (0%)	
Wildtype	19 (83%)	17 (71%)	
Unknown	4 (17%)	7 (29%)	
MGMT promotor methylation			0.347
Methylated	6 (26%)	7 (29%)	
Unmethylated	13 (57%)	9 (38%)	
Unknown	4 (17%)	8 (33%)	
Adjuvant therapy			0.148
None	3 (13%)	2 (8%)	
Chemo or radiotherapy	3 (13%)	0 (0%)	
Chemoradiation*	17 (74%)	22 (92%)	
NIHSS post-operative, median (IQR)	0 (0-2)	0 (0-2)	0.825
KPS after surgery, median (IQR)			
Seven weeks	90 (90-100)	90 (80-100)	0.412
Three months	90 (80-100)	90 (70-100)	0.540
Six months	90 (70-90)	70 (60-90)	0.228
Quality of life change, baseline *vs.* six months**			
Global health status	-2 (35)	-14 (28)	0.344
Physical functioning	-8 (31)	-13 (18)	0.267
Cognitive functioning	-11 (32)	-2 (30)	0.893
Motor dysfunction	2 (21)	5 (20)	0.893
Communication deficit	1 (26)	-6 (22)	0.609
Overall survival, median (95% CI), days	377 (247-507)	372 (320-424)	0.751
Progression-free survival, median (95% CI), days	227 (107-347)	233 (153-313)	0.937

Data are n or n (%), unless stated otherwise. *Stupp protocol **A change of ≥10 points is considered to be clinically relevant.

KPS, Karnofsky performance status; IDH, isocitrate dehydrogenase; MGMT, methylguanine; DNA, methyltransferase; NIHSS, National Institutes of Health stroke score.

Intraoperative ultrasound was used four times (range two to nine) on average per surgery. In the operating room, surgeons estimated that complete tumor resection was achieved in 15 (65%) of 23 patients when intraoperative ultrasound was used and in 17 (71%) of 23 patients without the use of intraoperative ultrasound (p=0.680). However, cases in which complete resection was thought to be achieved corresponded with radiological complete resection in only two (11.8%) of 17 in the standard surgery group and in seven (46.7%) of 15 patients in the intraoperative ultrasound group (proportion difference 34.9%, 95% CI 3.5-59.6, p=0.031; odds ratio 6.6, 95% CI 1.1-39.3, p=0.049). Median surgery time with intraoperative ultrasound guided surgery (177 minutes, IQR 135-255) was comparable to standard surgery (179 minutes, IQR 146-227, p=0.907).

Secondary outcome in terms of overall survival, progression free survival and health-related quality of life did not differ between treatment arms (p>0.05, details available as **Supplemental Material**). Median Karnofsky performance status seven weeks and three months after surgery was 90 (IQR 70/80-100) in both treatment groups. Six months after surgery, Karnofsky performance status was 60 or below in three patients (17%) who underwent intraoperative ultrasound surgery and in seven patients (37%) who underwent standard surgery (p=0.269). Neurological outcome as measured using the NIHSS scale within one week after surgery did not significantly differ between treatment groups (NIHSS 0 (IQR 0-2), p=0.825). In the intraoperative ultrasound *vs.* standard surgery groups, 16 (70%) respectively 19 (79%) patients had the same neurological status on the NIHSS scale after surgery as before surgery and five (22%) respectively three (13%) patients had neurological improvement. Four (9%) of all 47 patients had new or worsened neurological deficits: two (8%) patients who underwent intraoperative ultrasound guided surgery (one patient with hemiparesis and one with delirium and superior sagittal sinus thrombosis) and two patients (8%) who underwent standard surgery (one patient with aphasia and one with postoperative hemorrhage). Characteristics of these patients are presented in more detail in **Table 4**. Frequency of new or worsened neurological deficits did not significantly differ between treatment groups (p=0.591).

**Table 4:** Details of patients with complications.

Patient number	Sex	Age	Treatment group	NIHSS before surgery	NIHSS after surgery	CTCAE grade	Details of complication	Treatment of complication	KPS Seven weeks-three months-six months	Survival (days)
1	Male	75	Resection under standard neuronavigation	1	5	2	Aphasia	None	80-80-70	377
7	Male	65	Resection under standard neuronavigation	0	2	4	Postoperative hemorrhage	Emergency craniotomy	80-80-60	361
30	Male	59	Intraoperative ultrasound	1	9	2	Delirium and a sagittal sinus thrombosis	Haldol for delirium; Fraxiparine for thrombosis	n/a	36
35	Male	43	Intraoperative ultrasound	0	14	3	Left sided hemiparesis and central facial palsy	None	50-40-n/a	172

NIHSS, National Institutes of Health Stroke Scale; CTCAE, Common Terminology Criteria for Adverse Events grading v.4.0; KPS, Karnofsky Performance Status; n/a, not applicable.

## Discussion

This is the first randomized controlled trial that assessed the value of intraoperative B-mode ultrasound guided surgery on the extent of glioblastoma resection. Our trial showed that intraoperative B-mode ultrasound guided surgery enables complete contrast-enhancing tumor resections more often than standard surgery, without harming patients in terms of neurological outcome, functional performance or health-related quality of life.

Complete resection of contrast-enhancing tumor during glioblastoma surgery has consistently been associated with longer overall survival (2, 3, 23, 24). It is shown that 5-aminolevulinic acid and intraoperative MRI guided surgery improves the extent of glioblastoma resection (4–6, 25–27).

An alternative potentially cost- and time-effective technology that is used to acquire real-time imaging and apply brain shift correction during neuro-oncological surgery is intraoperative ultrasound guidance (7). Retrospective studies have suggested that intraoperative ultrasound increase the extent of tumor resection during surgery (8–10). These studies however, included different glioma subtypes and held different definitions of gross total resection, thereby introducing some degree of selection and confounding biases. No randomized controlled trial was performed to date to assess the value of intraoperative ultrasound to maximize the extent of glioblastoma resection (4).

When compared to intraoperative MRI, intraoperative ultrasound has two advantages; it is less expensive and, as shown in our trial, it does not prolong surgery time. Surgeons could rapidly control for residual tumor in the resection cavity multiple times during surgery without prolonging surgery time. The interpretation of intraoperative ultrasound images might be more challenging than intraoperative MRI images, however, the integration of an intraoperative ultrasound systems with a standard neuronavigation system (as used in our trial) enables surgeons to overlay intraoperative ultrasound images on navigational preoperative MRI scans, which may facilitate the interpretation of ultrasound images and consequently the accuracy of complete tumor resection estimation. Importantly, we observed that when intraoperative ultrasound was used, surgeons were able to estimate complete tumor resection in the operating room significantly more accurately than with standard surgery without ultrasound guidance, as confirmed on post-operative MRI.

Complete tumor resection as a primary outcome has some aspects that need careful consideration. Several definitions of complete tumor resection exist across studies, both qualitatively as quantitatively (2, 25). Studies defined complete tumor resection as no residual contrast-enhancing tumor on a post-operative MRI scan (24, 28), which is a relatively stringent definition (if quantitatively assessed) and may result in false positive assessment of the presence of residual tumor due to non-specific contrast enhancement such as ischemia, small vessels, a non-specific tissue response, or by T1-hyperintense blood in the surgical cavity that is incorrectly interpreted as enhancement. In our trial, this was mitigated by overlaying the identically acquired and registered pre- and post-contrast T1w sequences to exclude any T1-hyperintense areas from the residual tumor delineation. To take interpretation varieties into account, some studies defined complete tumor resection as contrast-enhancing residual tumor smaller than 0.175 cm^3^ following Stummer et al. (5, 6) while others have used extent of resection cut-off percentages, such as 95%, 97% or 98% (27, 29–32). In relation to this, it is known that residual tumor assessment of glioblastoma has a low interobserver agreement, introducing some degree of subjectivity when distinguishing contrast-enhancing residual tumor from non-specific contrast enhancement (33). In this trial, complete tumor resection was defined as more than 99% resection of contrast-enhancing tumor volume, accepting residual contrast-enhancing volume smaller than one percent to account for the non-tumor related post-surgical reactive enhancement amongst others, which is present even on early (within 48h) post-operative MRI scans (34). Even then, our complete resection proportion is lower than that reported in conventional treatment arms of previous trials, however the median extent of resection in both groups were high (97% in intraoperative ultrasound *vs.* in 95% standard surgery) (5, 6). This indicates that the low proportion of complete resection could partially be explained by a possible stringent interpretation of small contrast-enhancing voxels in the surgical cavity rather than surgical performance, as described earlier. This may have led to false positive interpretation of residual contrast enhanced tumor (i.e. false negative complete resection outcomes) in our trial. Importantly, we included only glioblastoma that were deemed complete resectable prior to surgery, which could partially explain the high median extent of resection percentages in both treatment groups (97% with ultrasound guidance and 95% with standard surgery). Our hypothesis in this trial was that in these totally resectable deemed glioblastoma, intraoperative ultrasound would be useful to resect the last small tumor portions and thus to actually achieve complete resection more often. As mentioned earlier, since only high resection cut-off percentages (e.g. >97% and >98%) (25, 28) are associated with survival benefit, we chose complete resection, rather than resection percentage as primary outcome.

A limitation of this trial is that it was not double-blinded, however, complete resection of contrast-enhancing tumor, our primary outcome, was assessed by an independent, blinded neuroradiologist.

Another limitation of this trial is that only 2-D, B-mode intraoperative ultrasound imaging was used in our trial without the use of advanced ultrasound techniques. Earlier studies have shown that intraoperative B-mode ultrasound enables gross total resection of contrast enhancing tumor more frequently (8–10). Tumor detection however, is dependent on factors such as surgeon experience, resolution and used probe. As Coburger et al. showed, a linear array ultrasound probe is superior in detecting tumor than conventional ultrasound probe. This is even more relevant, since it is shown that the detection of especially smaller residual volumes (<1cm^3^) becomes a challenge. Advanced ultrasound techniques such as contrast enhanced ultrasound, Doppler ultrasound and elastography have the potential to improve tumor detection during surgery (11). Prada et al. showed that next to B-mode imaging, contrast enhanced ultrasound to be useful and highly specific in the identification of residual tumor (12, 13). Finally, it is also shown that elastography could better discriminate between different tissues and was able to identify lesion margins sharper compared to B-mode (14). Future studies that include such advanced ultrasound techniques to study the potential of supporting the surgeon to safely maximize the extent of glioblastoma resection are desired. Our trial did not show any overall survival benefit for patients who underwent intraoperative ultrasound. It should be noted however, that our trial did not aim and was not designed or powered to show differences in overall survival. Although complete glioblastoma resection is associated with survival benefit, future trials on image guidance with a suited design and larger sample size are still needed to show any potential clinical benefit directly in the trial itself for patients with glioblastoma.

In conclusion, this randomized controlled trial showed that intraoperative ultrasound guided surgery enables complete contrast-enhancing tumor resections more often than standard surgery, without harming patients in terms of neurological outcome, functional performance or health-related quality of life. Intraoperative ultrasound is a safe and useful intraoperative imaging alternative and, just as intraoperative MRI or 5-aminolevulinic acid guided surgery, can be considered to maximize the extent of contrast-enhancing glioblastoma resection.

## Data Availability Statement

The original contributions presented in the study are included in the article/**Supplementary Materials**, further inquiries can be directed to the corresponding author.

## Ethics Statement

The studies involving human participants were reviewed and approved by Medical Ethical Committee of Erasmus MC (MEC-2015-46). The patients/participants provided their written informed consent to participate in this study.

## Author Contributions

Study design, clinical data collection: FI and AV. Primary outcome assessor: MS. Data interpretation: all authors. All authors contributed to the article and approved the submitted version.

## Funding

This study was supported by a grant from the Coolsingel Foundation (project number 105517), The Netherlands. The funders had no role in the design, execution or writing of this trial.

## Conflict of Interest

MS reports an honorarium received from Parexel Ltd (paid to institution) and speaker fees from GE Healthcare (paid to institution).

The remaining authors declare that the research was conducted in the absence of any commercial or financial relationships that could be construed as a potential conflict of interest.
