# Supplementary figures and images for: Intraoperative B-Mode Ultrasound Guided Surgery and the Extent of Glioblastoma Resection: A Randomized Controlled Trial
Source: Front Oncol. 2021 May 19;11:649797. doi: 10.3389/fonc.2021.649797 (PMC8170308; doi:10.3389/fonc.2021.649797)

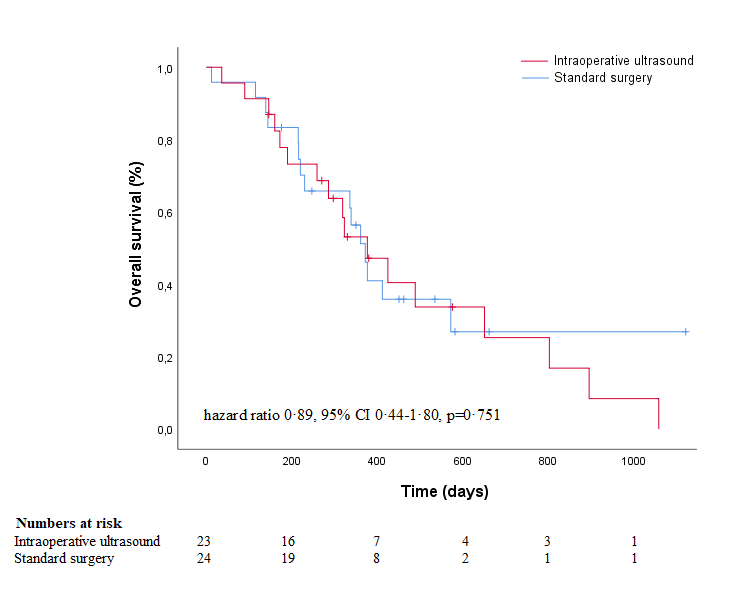

Supplement: Supplementary file 2 [file Image_1.tif]
